# Endothelial dysfunction markers and immune response indices in cosmonauts’ blood after long-duration space flights

**DOI:** 10.1038/s41526-022-00237-0

**Published:** 2022-11-02

**Authors:** D. S. Kuzichkin, I. A. Nichiporuk, O. A. Zhuravleva, A. A. Markin, M. P. Rykova, T. V. Zhuravleva, A. A. Sadova, O. V. Kutko, V. A. Shmarov, S. A. Ponomarev

**Affiliations:** State Scientific Center of the Russian Federation - Institute for Biomedical Problems of the Russian Academy of Sciences, Moscow, Russia

**Keywords:** Physiology, Cytokines, Interleukins, Risk factors, Prognostic markers

## Abstract

Space flight factors are known to cause a malfunction in the human immune system and lead to damage to blood vessels. The hemostatic function of endothelium during space missions and its interaction with human immunity has not been determined so far. In this work, we investigated the markers of endothelial activation and damage (plasma concentrations of soluble thrombomodulin fraction (sTM), von Willebrand factor (vWF), highly sensitive C-reactive protein (hs-CRP)), as well as the level of D-dimer and compared them to the immunological parameters characterizing the state of human humoral and cellular immunity. The immune status of long-duration ISS crewmembers was assessed by whole-blood testing, and comprehensive postflight immune assessment included the analysis of leukocyte distribution. Flow cytometry was applied to determine the absolute counts and the percentage of lymphocyte subsets: B cells (CD19^+^), T cells (CD3^+^, CD3^+^CD4^+^, CD3^+^CD8^+^), NK cells (CD3^−^CD16^+^CD56^+^, CD11b^+^CD56^+^), and activated subsets (CD3^+^CD25^+^ and CD3^+^HLA-DR^+^). The in vitro basal cytokine production was investigated in whole blood cell culture. The cytokines IFN-gamma, IL-1-beta, IL-4, IL-6, IL-10, IL-18, and TNF-alpha were measured in plasma and the 24-h supernatants by a sensitive enzyme-linked immunosorbent assay. A significant increase in the plasma levels of vWF and hs-CRP and a decrease in the concentration of sTM after spaceflights were detected. Divergent changes in the parameters characterizing the state of the immune system were observed. We propose that the changes revealed may lead to an increase in the procoagulant activity of blood plasma, suppression of protein C activation and thrombin inhibition, as well as to an increase in the adhesive-aggregate potential of platelets, especially in case of changes in the rheological characteristics of blood flow during re-adaptation to ground conditions. We also speculate that the immune system might play an important role in vessel damage during long-duration missions.

## Introduction

It is known that the effect of space flight factors on the human body can lead to damage, activation, and increasing dysfunction of endothelium^1^. It has been shown that the number of endothelial cells circulating in the blood increases after 2 months of head-down tilt bed-rest^2^. Signs of cytoskeleton damage increased permeability of cell membranes, and a decrease in the proliferative activity of cultured endothelial cells after orbital flight were revealed^3^. However, the function of the endothelium consisting in regulating the blood aggregate state under the influence of space flight factors has not been practically studied. It is common knowledge that factors of space flight can damage blood vessels and affect the blood hemostasis system^4^, increasing the risk of thrombophilia and, in some cases, leading to the appearance of occlusive thrombosis during orbital flight^5^. We selected two specific markers of endothelial hemostatic potential for research, namely, von Willebrand factor (vWF) and thrombomodulin (TM), as well as highly sensitive C-reactive protein (hs-CRP) as an additional marker of endothelial dysfunction^6^. vWF is a multimeric glycoprotein present in blood plasma, endothelial cells, megakaryocytes, and platelets. vWF is constantly secreted into the bloodstream by the endothelial cells’ Weibel–Palade bodies and by the platelets’α-granules during activation. The pool of circulating vWF consists of multimers of various sizes, from several dimers to high-molecular multimers, which contain 11–20 dimers. The former mainly serve as carriers (apoenzymes) of the factor VIII coagulation cascade, protecting it from proteolysis^7^, and the latter mediates platelet adhesion and aggregation^8^. TM is synthesized mainly in endothelial cells and is expressed on their membrane. It regulates the activity of thrombin and physiological anticoagulant protein C^9^. The presence of soluble TM fragments in plasma is sometimes considered a sign of endothelial damage, although it is also functional in plasma: it serves as a cofactor for thrombin binding and protein C activation^10^ and mediates antifibrinolytic activity^11^. Hs-CRP in the form of a protein made of identical subunits forming a cyclic pentamer is synthesized by hepatocytes, adipocytes, and atherosclerotic plaques. It enhances the synthesis of reactive oxygen species, binds to oxidized particles of low-density lipoproteins (LDL), and stimulates their absorption by macrophages, contributing to an increase in the production of adhesion molecules (ICAM, VCAM, E-selectin, MCP-1). It induces the secretion of monocyte tissue factor (TF) and inhibits the production of plasminogen activator I inhibitor (PAI–I) and tissue plasminogen activator (TPA). This protein has a direct effect on the release of proinflammatory cytokines from leukocytes, thereby disrupting the vasoreactivity of the endothelium and causing instability of atherosclerotic plaques, which, in turn, leads to an increased risk of thrombosis^12^.

The aim of this work was to study the levels of vWF, TM, and hs-CRP in cosmonauts’ blood plasma after long-term orbital expeditions to the International Space Station and to compare the results to the parameters characterize the state of the immune system which can be involved in into endothelial dysfunction.

## Results and discussion

### The dynamics of the parameters in the post-flight period

**Fig. 1:**
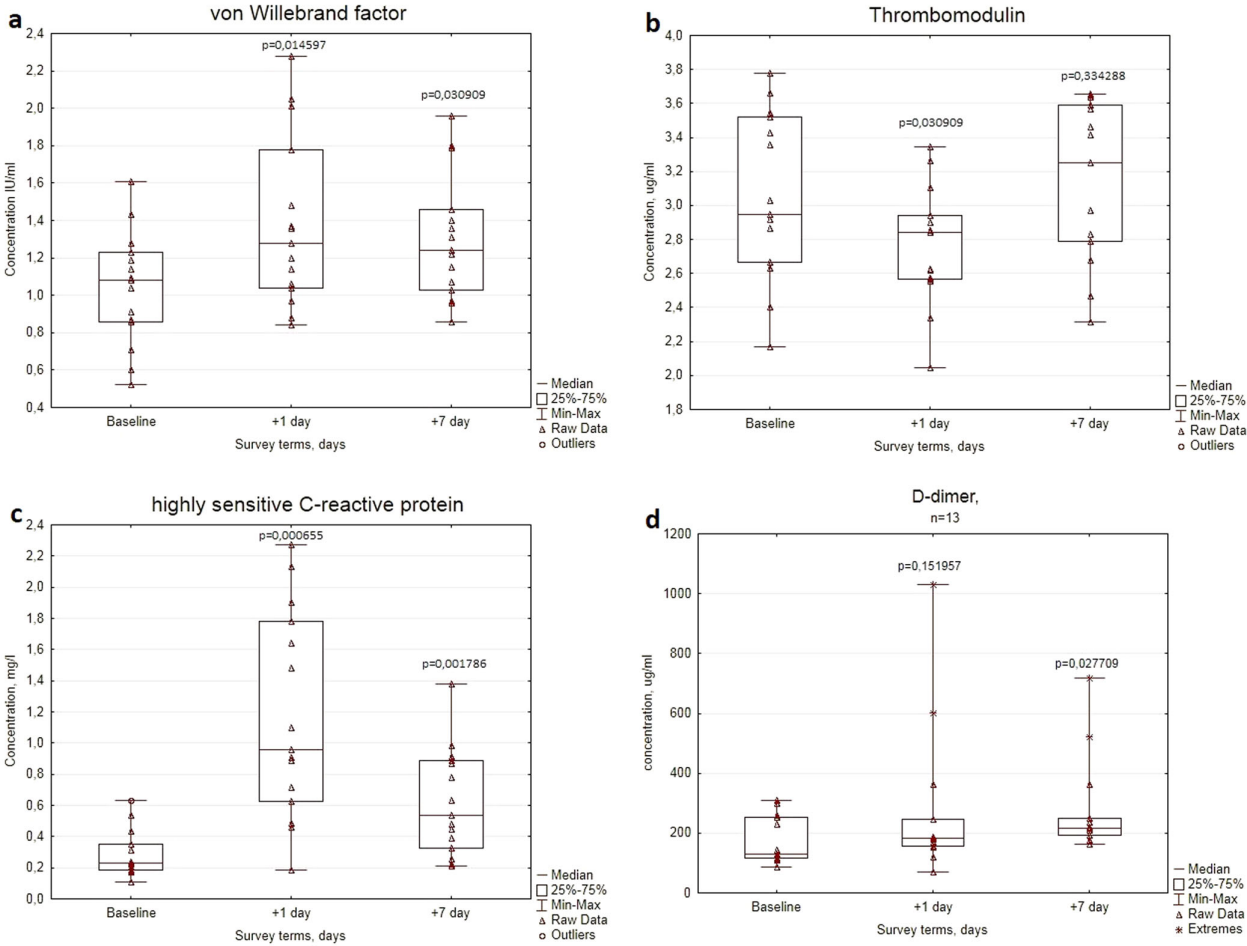
The concentrations of the blood plasma parameters of cosmonauts before the mission (Baseline) and after space flight completion (Days +1 and +7). **a** von Willebrand factor, **b** thrombomodulin, **c** highly sensitive C-reactive protein, and **d** D-dimer. The center lines represent the median, the bounds of boxes represent the 25th percentile and 75th percentile (Q1 and Q2 correspondingly), the bonds of whiskers represent the minimum and the maximum values, the triangles within the diagrams are the raw data for particular volunteers, *p* values were obtained using the Wilcoxon’s test.

**Fig. 2:**
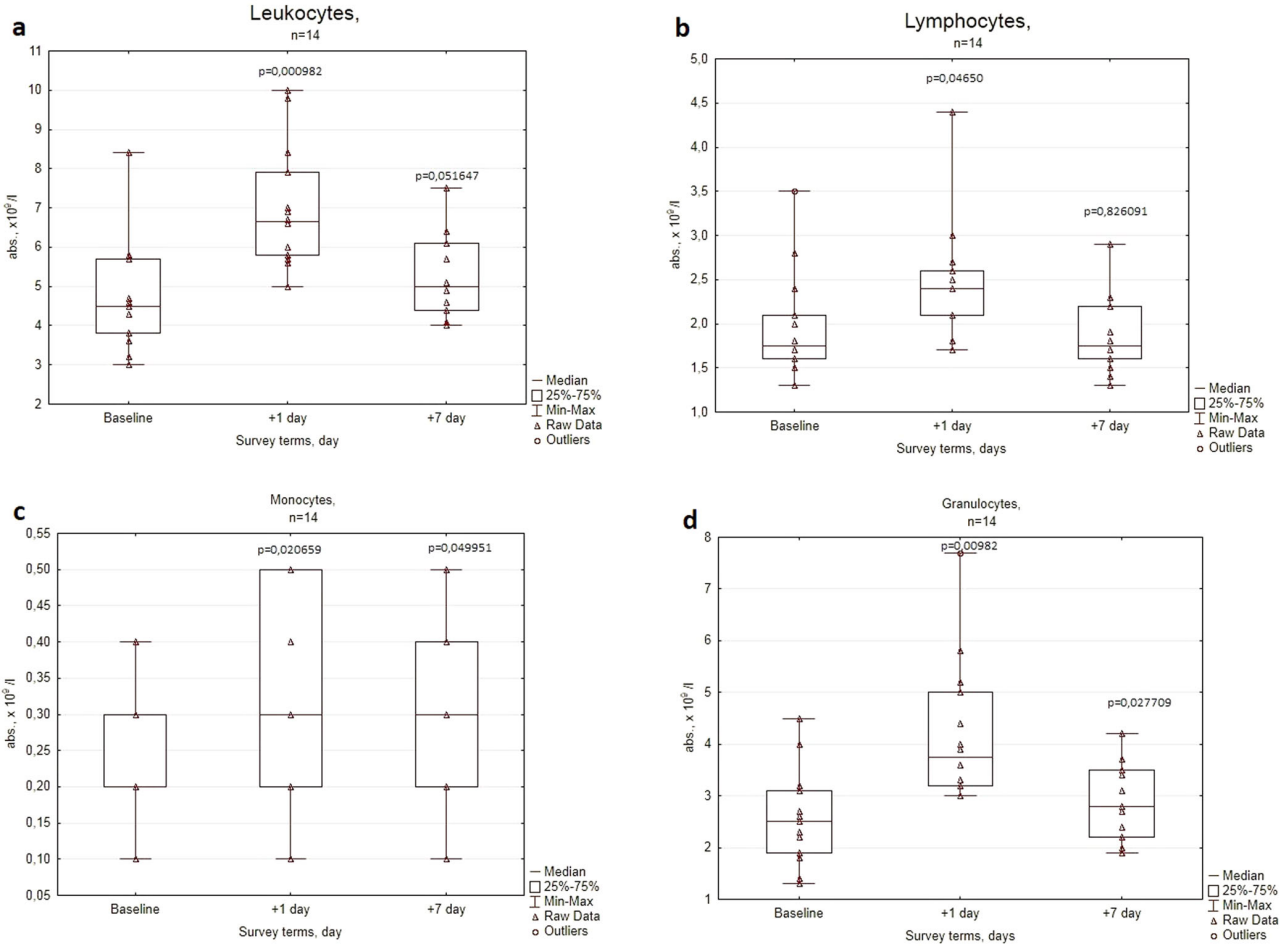
Cosmonauts’ leukograms before (Baseline) and after space missions (Days + 1 and +7). The concentrations of the following cell populations are represented: **a** leukocytes, **b** lymphocytes, **c** monocytes, **d** granulocytes. The center lines represent the median, the bounds of boxes represent the 25th percentile and 75th percentile (Q1 and Q2 correspondingly), the bonds of whiskers represent the minimum and the maximum values, the triangles within the diagrams are the raw data for particular volunteers, *p* values were obtained using the Wilcoxon’s test.

**Fig. 3:**
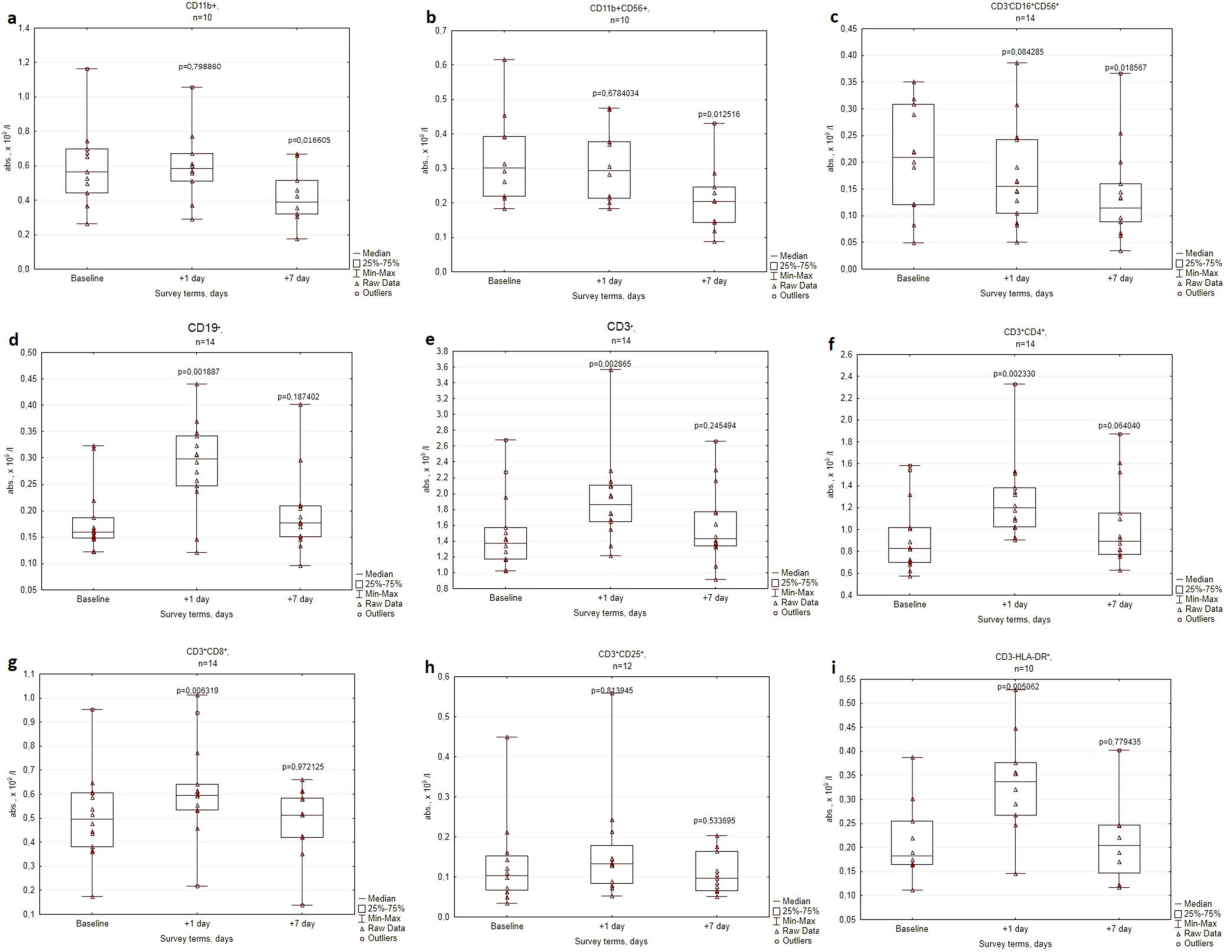
Lymphocyte subpopulations in peripheral blood of cosmonauts before (Baseline) and after spaceflights (Days + 1 and +7). **a** CD11b^+^, **b** CD11b^+^CD56^+^, **c** CD3^-^CD16^+^CD56^+^, **d** CD19^+^, **e** CD3^+^, **f** CD3^+^CD4^+^, **g** CD3^+^CD8^+^, **h** CD3^+^CD25^+^, **i** CD3^-^HLA-DR^+^. The center lines represent the median, the bounds of boxes represent the 25th percentile and 75th percentile (Q1 and Q2 correspondingly), the bonds of whiskers represent the minimum and the maximum values, the triangles within the diagrams are the raw data for particular volunteers, *p* values were obtained using the Wilcoxon’s test.

Parameters of vWF, TM, and hs-CRP in the dynamics of pre- and postflight examinations of cosmonauts are shown in Figs. 1–3. The concentrations of vWF in analyzed blood plasma samples were significantly increased by (*p* = 0.015) on the first day after the flight and by (*p* = 0.031) on the 7th day of the recovery period. D-dimer concentration was significantly elevated (*p* = 0.028) in the recovery period (Fig. 1).

The concentration of TM was moderately (*p* = 0.031) reduced on the 1st day after flight. On the 7th day of the recovery period, the plasma level of TM did not significantly differ from the baseline (Fig. 1).

In the examined ISS crewmembers statistically significant peripheral leukocyte subset changes on the 1st day after landing (compared to preflight baseline values) included upregulated absolute leukocyte, monocyte, granulocyte, and lymphocyte levels, and decreased lymphocyte percentage (Fig. 2, Table 2). There was also a significant reduction in the percentage of circulating NK cells, whereas the levels of B cells, T cells, CD4, and CD8 subsets were elevated. Interestingly, among constitutively activated T-cell subsets, the disparity was observed in the CD25+ and HLA-DR+ subsets. The level of CD25+ T cells remained unchanged, while the level of activated T cells (HLA-DR+) was elevated (Figs. 2, 3; Table 1).

**Table 1:** Peripheral leukocyte subset in the dynamics of pre- and postflight examinations of cosmonauts (median; q25–q75), *p* values were obtained using the Wilcoxon’s test.

Parameter		Baseline	+1 day	+7 day
Lymphocytes	% in leukocytes	42,9; 40,8–45,4	36,3; 32,3–42,6*	36,5; 33,6–42,5*
Monocytes	% in leukocytes	4,6; 4,3–5,0	4,5; 3,7–5,0	5,9; 5,2–6,5*
Granulocytes	% in leukocytes	52,5; 49,1–55,3	58,5; 53,1–62,7*	56,6; 51,1–59,4
CD11b^+^	% in lymphocytes	28,0; 25,5–34,5	24,0; 21,8–25,8*	24,0; 18,5–26,0*
CD11b^+^CD56^+^	% in lymphocytes	15,5; 13,3–17,0	11,5; 10,2–13,5*	11,0; 7,5–13,0*
CD3^−^CD16^+^CD56^+^	% in lymphocytes	11,0; 8,3–12,8	6,5; 4,5–9,0*	6,5; 5,0–7,8*
CD19^+^	% in lymphocytes	9,5; 9,0–11,0	12,0; 10,5–14,0*	10,5; 7,3–12,0
CD3^+^	% in lymphocytes	76,5; 73,0–78,0	78,5; 75,5–81,8*	80,0; 77,5–82,5*
CD3^+^CD4^+^	% in lymphocytes	47,0; 43,3–53,0	51,5; 48,5–54,5*	51,5; 46,5–56,8*
CD3^+^CD8^+^	% in lymphocytes	25,0; 22,3–27,8	24,0; 22,0–28,3	24,0; 23,0–26,0
CD3^+^CD25^+^	% in lymphocytes	6,0; 3,8–6,8	5,5; 3,0–8,3	6,0; 3,0–6,5
CD3^+^HLA-DR^+^	% in lymphocytes	11,0; 10,0–12,8	14,0; 12,0–15,0*	13,0; 9,8–14,0

^*^Significant difference (*p* < 0.05).

In this study, no significant alterations were observed in the plasma concentration of inflammatory or adaptive/T-regulatory cytokines (TNFα, IL-1b, IL-6, IL-8, IFN-γ, IL-18, IL-4, IL-10) upon the completion of spaceflights (Table 2). At the same time, the basal production of TNFα and IL-8 by cultured non-stimulated whole blood cells was significantly decreased, whereas the alterations in the basal production of other cytokines did not reach statistical significance.

**Table 2:** Cytokine levels in serum and culture supernatants in the dynamics of pre- and postflight examinations of cosmonauts (median; q25–q75), *p* values were obtained using the Wilcoxon’s test.

Cytokine (pg/mL)	Blood serum			Whole blood culture supernatants without activation		
	Baseline	+1 day	+7 day	Baseline	+1 day	+7 day
IL-1b	1,8; 1,6-4,0	1,8; 1,2-2,2	1,8; 0,3-2,0	5,9; 4,2-12,0	7,0; 6,6-8,8	6,0; 3,0-7,0
TNFα	0,1; 0,0-0,4	0,3; 0,1-0,4	0,6; 0,0-0,8	4,4; 1,9-13,2	2,4; 1,0-2,8*	1,7; 1,7-3,8
IL-6	0,7; 0,3-1,1	2,2; 0,2-3,0	0,8; 0,5-2,8	150,8; 30,2-229,1	3,2; 2,0-31,1	7,9; 6,3-21,3
IL-8	9,3; 5,3-14,1	16,2; 3,3-20,1	17,4; 16,4-18,5	1366; 79-2974	49; 24-185*	54; 52-438
INF-γ	3,7; 3,7-4,9	4,3; 0,0-5,5	3,7; 3,1-4,3	2,8; 1,3-6,1	3,1; 2,5-4,0	2,8; 2,8-4,3
IL-4	3,7; 3,7-4,9	4,3; 0,0-5,5	3,7; 3,1-4,3	2,8; 1,3-6,1	3,1; 2,5-4,0	2,8; 2,8-4,3
IL-10	6,8; 3,7-7,4	4,9; 4,9-14,2	6,8; 4,3-7,4	4,0; 1,4-7,1	3,7; 3,4-8,6	8,3; 4,3-11,1
IL-18	60,8; 50,7-121,4	80,2; 74,0-82,2	105,5; 84,9-159,8	10,5; 10,3-22,3	17,5; 16,4-28,9	21,7; 13,5-24,2

^*^Significant difference (*p* < 0.05).

During all examination timepoints the concentrations of hs-CRP in the cosmonauts’ blood were within the reference values for asymptomatic cohorts with low pre-test probability (0–2 mg/L^6^). But the significant variability of changes in C-reactive protein concentrations measured in a highly sensitive range drew our attention. For instance, on the 1st day after landing the level of CRP exceeded the pre-flight level by more than 4 times (*p* = 0.0007), and on the 7th day of the recovery period, its content in the cosmonauts’ blood remained 2.3 times higher compared to baseline (*p* = 0.0018) (Fig. 1).

### Multiple regression analyses

At the final stage of our research, the multiple regression analyses of cytokines and the parameters of hemostasis as independent variables in cosmonauts before and after space flights were done using the forward stepwise regression method with sTM and vWF as dependent variables (Table 3). The interpretation of these results shows that in more than 83% of all cases cosmonauts with higher levels of sTM had higher concentrations of serum IL-8 and IFN-γ, increased basal production of IL-8 and IL-10, and lower concentrations of IL-6 and hs-CRP in serum, which were accompanied by higher values of D-dimer (Table 3A). In turn, the results of the analysis evidence that higher vWF concentration in blood plasma in more than 70% of all cases was in direct correlation with serum IL-6 and basal production of IL-4, and it negatively correlated with basal production of IL-8 and serum IL-10 (Table 3B).

**Table 3:** Results of multiple regression analyses of cytokines and parameters of hemostasis in cosmonauts before and after space flights (*k* = 27; forward stepwise regression, dependent variables sTM and vWF).

(A) Dependent variable: sTM; *R* = 0.915; *R*^2^ = 0.837; adjusted *R*^2^ = 0.717 *F*(14, 19) = 6.969; *p* < 0.0001; Standard error of estimate: 184.1			
Independent variable	BETA	Standard error of BETA	*p*
D-dimer	0.741	0.150	<0.001
IL-6 in serum	–1.305	0.267	<0.001
Basal production of IL-10	0.647	0.139	<0.001
INF-γ in serum	0.718	0.152	<0.001
Basal production of IL-8	0.456	0.184	0.023
IL-8 in serum	0.356	0.151	0.029
hs-CRP	–0.345	0.157	0.041

(B) Dependent variable: vWF; *R* = 0.841; *R*^2^ = 0.708; adjusted *R*^2^ = 0.581 *F*(10, 23) = 5.576; *p* < 0.0003; Standard error of estimate: 0.145			
IL-6 in serum	0.413	0.160	0.017
IL-10 in serum	–0.523	0.143	0.001
Basal production of IL-8	−0.576	0.205	0.010
Basal production of IL-4	0.503	0.194	0.016

*R*—coefficient of multiple regression, *R*^2^—coefficient of multiple determination, *F* = regression mean square/residual mean square, BETA—standardized regression coefficient showing relative contribution of each indicated independent variable in the prediction of the dependent variable, *k*—number of degrees of freedom.

### The causes and the consequences of the changes observed

Taking into account that vWF is mainly secreted by the endothelium, the increase in its plasma level might occur due to damage to the endothelium and the release of this protein into the bloodstream^13^. The increase in the D-dimer concentration observed in the recovery period points to the activation of plasmin and the coagulation cascade during the descent from the orbit and the period of acute re-adaptation to Earth conditions, as plasma D-dimer levels reflect the intensity of fibrine formation and fibrinolysis several days past the acute incidence, in this case, past the endothelium damage. It is confirmed by the fact that the majority of the examined cosmonauts had hemorrhages in the form of petechiae or subcutaneous ecchymoses after the flight’s completion.

Probably, the increase in the level of synthesis and secretion of vWF was also observed due to the influence of mechanical and humoral stimuli on the intact endothelium. Among the mechanical stimuli, the most important one is the hydrodynamic pressure determined by the value of the directly proportional blood viscosity and the velocity gradient (shear rate) between the layers of the laminar blood flow (shear stress)^14^. The transformation of the mechanical stimulus of the shear stress into biological signals that control the functions of the endothelium is achieved by mechanotransduction^15^. The signal of shear stress changes perceived by the glycocalyx is transmitted to endothelial cells through the pathways including integrins, tyrosine kinase receptors, G-protein-bound receptors, ion channels and connective proteins, caveoles, and membrane lipid rafts^16,17^.

It has been well-cited that the hemostatic system and its components and the immune system are intricately related, with the two systems complementing each other to provide host defense and limit the dissemination of invading pathogens. Circumstantial evidence suggests that the innate immune system and coagulation system share a common evolutionary origin, which explains the extensive crosstalk between inflammatory cytokines and coagulation factors, with many components being important for both systems^18^. Endothelial cells (ECs) also actively participate in adaptive immune responses. So, ECs can modulate the metabolic reprogramming of T cells through the secretion of various signaling molecules. Similarly, several studies indicate that T cells play significant roles as regulators of ECs functions during inflammation through the secretion of many immunomodulatory molecules and cytokines^19^.

Interleukin-6 (Il-6) in combination with the soluble Il-6 receptor, interleukin-8 (Il-8), and tumor necrosis factor-α (TNF-α) significantly stimulates the release of vWF from the endothelial cells’ Weibel–Palade bodies. Il-6 prevents the cleavage of vWF by the plasma metalloproteinase ADAMTS-13^20^. An increase in the plasma level of Il-8 during the flight was shown, but no change in the concentration of Il-6 in blood plasma during and after the completion of the expedition was detected^21^. The level of TM expression can also be regulated by cytokines. It is known that interleukin-1β (Il-1β) suppresses TM mRNA^22^, and during space flight, an increase in the level of Il-1β is observed^23^. Thus, pro-inflammatory cytokines can increase the procoagulant potential and reduce the anticoagulant potential of the endothelium. In addition to this, the increased activation of the adaptive immune system and the uncontrolled release of cytokines by immune cells could be regarded as one of the risk factors for the development of endothelial dysfunction during a space flight. Among the first cells to secrete cytokines in response to pathogenic or harmful signals are immune cells^24^. Similar to the data of the previous long-term spaceflight observations^25,26^, despite the significant increase in the most of leukocyte subsets, the ability of immune cells to produce cytokines on the 1st day after landing was impaired.

On the other hand, vasopressin may be one of the reasons for the increased plasma vWF concentration, as it was shown to be upregulated during space flight^27^ and to stimulate the release of vWF from the endothelial cells’ Weibel–Palade bodies^28^.

The elevated plasma vWF concentration usually leads to the increase in the procoagulant potential of the coagulation cascade and the adhesive-aggregative properties of platelets, however, the vWF cofactor activity and its metabolism is largely determined by the value of the blood flow shift rate in the vessels^13,29,30^, as well as by the activity of ADAMTS-13 metalloprotease^31–33^.

In model experiments on small animals, it was shown that the intensity of platelet adhesion and aggregation was reduced after hypogravity and increased after hypergravity exposure^34^. The platelet activation after hypergravity exposure (15 min, 3 Gz) was also observed^35^.

Recent studies have shown that vWF is also involved in the processes of inflammation, linking thrombosis to inflammation. Inflammation can provoke thrombosis through the vWF-dependent pathway, which includes activation of the endothelium, secretion of vWF into the bloodstream, and interaction with platelets. vWF multimers and platelets attached to the damaged and activated endothelium can serve as places for recruiting white blood cells. Together, this predisposes to the spread of the inflammatory process^36,37^.

It should be noted that the increase in the level of vWF in plasma does not lead to the increase in the factor VIII level and does not affect the intensity of its cleavage by protein C, since vWF is always present in excess relative to the level of factor VIII^38^. However, if platelets are activated by vWF in the areas of high shear stress, they start releasing polyphosphates and contribute to the initiation of the internal pathway (contact phase) of the coagulation cascade^39^. The decrease in the plasma level of TM with a high probability indicates a decrease in the amount of its form immobilized on the endothelial membrane, since the damage of endothelium is accompanied by the destruction of membranes, and its activation leads to a decrease in the expression of anticoagulants. Thus, the above-mentioned changes should lead to an increase in procoagulant activity and a decrease in the anticoagulant activity of the plasma.

Lee et al. when examining 13 astronauts (10 men and three women aged 38–58) who participated in the ISS missions with a duration of 126–340 days, showed that the concentration of CRP in cosmonauts’ blood both on the day of landing and on the 5th day of the recovery period was higher than the preflight values. The authors explain the increased level of hs-CRP in the astronauts’ blood after flights by muscle injury during landing^40^.

Recently, cases of decreased blood flow and of occlusive thrombosis in ISS crewmembers during the orbital flight were reported^5^. The results of this study indicate that space flight factors cause the shift in the hemostasis balance towards coagulation up to the risk of thrombophilia development. However, the regulation of blood aggregate states under the influence of space flight factors is still poorly investigated due to the complications of conducting tests and measurements onboard a spacecraft. The recent review on space flight effects on venous blood clotting^41^ sheds little light on the investigations of post-flight blood coagulation. Indeed, one can find very little data on the hemostasis state after space missions and almost no results of onboard experiments or of studies of sample return capsules. They mostly study blood coagulation in ground-based model experiments. According to the results of long-term anti-orthostatic hypokinesia (20–60 days), thrombin and fibrin formation are upregulated together with increased fibrinolysis. The other review on thrombosis risks during and after space flights describes the experience of ultrasound analysis (UA) application for intravascular thrombin formation investigation and estimates the informativeness and objectiveness of such methods^42^. Generally, according to the results of UA and magnetic resonance imaging (MRI), as well as to the longstanding comprehensive analysis there were no signs of thrombotic structures formation observed, except for Marshall-Goebell K. (2019) reported the two cases of venous thromboembolia during the space flight. The results of hypokinesia and “dry” immersion experiments indicated a tendency to hypocoagulation during bed rest and hypercoagulation during the orthostatic probe, as well as the increase in the circulating endothelial microparticles during “dry” immersion^42^.

Earlier, our laboratory showed a significant (*p* < 0.05) increase in the potential activity of the internal pathway of the coagulation cascade (by activated partial thromboplastin time (APTT) shortening) in cosmonauts^4^. In the baseline period, the values of the interpersential (10–90%) range of activated partial thromboplastin time for cosmonauts were significantly higher than the average population reference values range. And on the 1st day after the flight, their significant decrease and convergence with the range of the average population reference values were observed. There were also corresponding exceedances of the average population range reference interval limits in terms of antithrombin III and protein C plasma level indices^43^.

APTT reflects the integral potential of the internal coagulation pathway. Its activation can be considered an evolutionarily developed mechanism that prepares the body for possible blood loss in extremely stressful conditions. In a group of cosmonauts, the reduced initial potential of the internal pathway may indicate the presence of adaptive changes in the body. These changes are aimed at preventing the threat of thrombophilia when the coagulation process is activated during training and or in case of exposure to stress factors of a space flight.

### Prospects

We conclude that the activation of procoagulant properties of endothelium by mechanical and humoral stimuli develops during a space flight and in the re-adaptation period. This effect may further cause coagulation cascade activation and stimulate thrombocytes’ adhesive and aggregative properties. Thus, the determination of hs-CRP serum level together with vWF and DD plasma level can be used as a marker of endothelial damage when an almost healthy person is exposed to extreme factors.

Overall, we can speculate that a large set of space flight-associated factors and physiological effects, which alter physiological characteristics, including blood rheology and concentration, psychological stress, and vessels damage, lead to the increase in the endothelium procoagulant potential and the activation of both plasma and cellular components of hemostasis. Moreover, immune mechanisms apparently have a particular role in these processes. On the other hand, upregulation of fibrin formation and platelets activation together with the increase in the concentration of adhesion molecules, namely, the von Willebrand factor, may in their turn affect inflammation and immune response development. This should be taken into account while developing countermeasures for long-duration space missions as well as in practical healthcare on Earth.

## Methods

The methods were performed in accordance with relevant guidelines and regulations approved at a meeting of the Academic Council and verified by the Biomedicine Ethics Committee of the RF SRC—Institute of Biomedical Problems, Russian Academy of Sciences (Physiology Section of the Russian Bioethics Committee Russian Federation National Commission for UNESCO).

### Blood sampling

The study involved 15 male cosmonauts aged 37–60 years, who performed orbital flights lasting from 115 to 205 days. All subjects provided written informed consent in accordance with the Declaration of Helsinki to take part in the experiments. Venous blood was taken in the baseline period 30–45 days before the start, as well as on the first and the seventh days of the recovery period. Each of the subjects participated only in one of the examination cycles. Blood sampling was carried out in the “Greinerbio” vacuum tubes “Vacuette” with a standard sodium citrate content (in the ratio of 9 blood volumes to 1 volume of 3.8% sodium citrate solution) and also in tubes without preserving agent. Citrated plasma as well as blood serum was obtained by centrifugation at 1800 × *g* for 10 min, and out of the citrated plasma the supernatant platelet-poor fraction was taken.

### Enzyme-linked immunosorbent assay and immunoturbidimetric assay

The concentrations of vWF and TM in the citrated plasma were determined by the standard ELISA method using test kits of Technoclone GmbH (Austria) and Hycult Biotech Inc. companies (USA). The concentration of hs-CRP was measured in blood serum by the immunoturbidimetric method on the “Biotecnika Instruments” (Italy) automatic biochemical analyzer “Targa BT 3000”. D-dimer level was measured by immunoturbidimetry using Sysmeх СA-1500 equipment and commercial kits (Siemens Healthineers, Germany).

### WBC count and immunophenotyping

Peripheral blood samples were obtained by antecubital venous puncture, collected in vacutainer tubes containing ethylenediaminetetraacetic acid (EDTA) as an anticoagulant (Vacuette®, Greiner bio-one, Kremsmünster, Austria), and processed by flow cytometry within two hours after sampling. Absolute and differential blood cell count was calculated for all samples using Hematology Analyzer MEK 6318 (Nihon Kohden, Japan).

For immunophenotyping commercially available fluorochrome-conjugated monoclonal antibodies (eBioscience, USA) were used at the manufacturer’s recommended concentrations: anti-CD45 (Clone 61D3, FITC, ref. no. 11-2459-42), anti-CD19 (Clone HIB19, PE, ref. no. 12-0199-80), anti-CD3 (Clone UCHT1, FITC, ref. no. 11-0038-42), anti-CD4 (Clone RPA-T4, PE, ref. no. 12-0049-42), anti-CD8 (Clone RPA-T8, PE, ref. no. 12-0088-80), anti-CD25 (Clone BC96, PE, ref. no. 12-0259-80), anti-CD11b (Clone ICRF44, FITC, ref. no. 11-0118-42), anti-CD16 (Clone eBioCB16 (CB16), PE, ref. no. 12-0168-42), anti-CD56 (Clone TULY56, PE, ref. no. 12-0566-42), anti-HLA-DR (Clone LN3, PE, ref. no. 12-9956-42), mouse IgG1 kappa isotype control (Clone P3.6.2.8.1, FITC, ref. no. 11-4714-81), and mouse IgG1 kappa isotype control (Clone P3.6.2.8.1, FITC and PE, ref. nos. 11-4714-81 and 12-4714-42). The used in this study monoclonal antibodies for each cosmonaut before and after space flight were purchased at the same time and were from the same lot number.

For peripheral blood analysis, 100 μL of blood was added to the appropriate tubes, and cells were processed according to the manufacturer’s instructions. In brief, blood was incubated with the antibodies for 20 min in the dark, followed by red blood cell lysis using OptiLyse B lysing solution (Beckman Coulter, USA) for 15 min in the dark. Cells were then washed twice in PBS and fixed in 200 mL of IOTest3 Fixative Solution (Beckman Coulter, USA).

The stained cells were analyzed on the FACSCalibur flow cytometer (Becton Dickinson, USA) using CellQuest software for data collection and analysis. 25,000 events were analyzed per tube. Isotypic controls were used for each assay to determine nonspecific staining. The fluorescence compensation was performed using CaliBRITE beads (Becton Dickinson, USA) and FACSComp software.

**Fig. 4:**
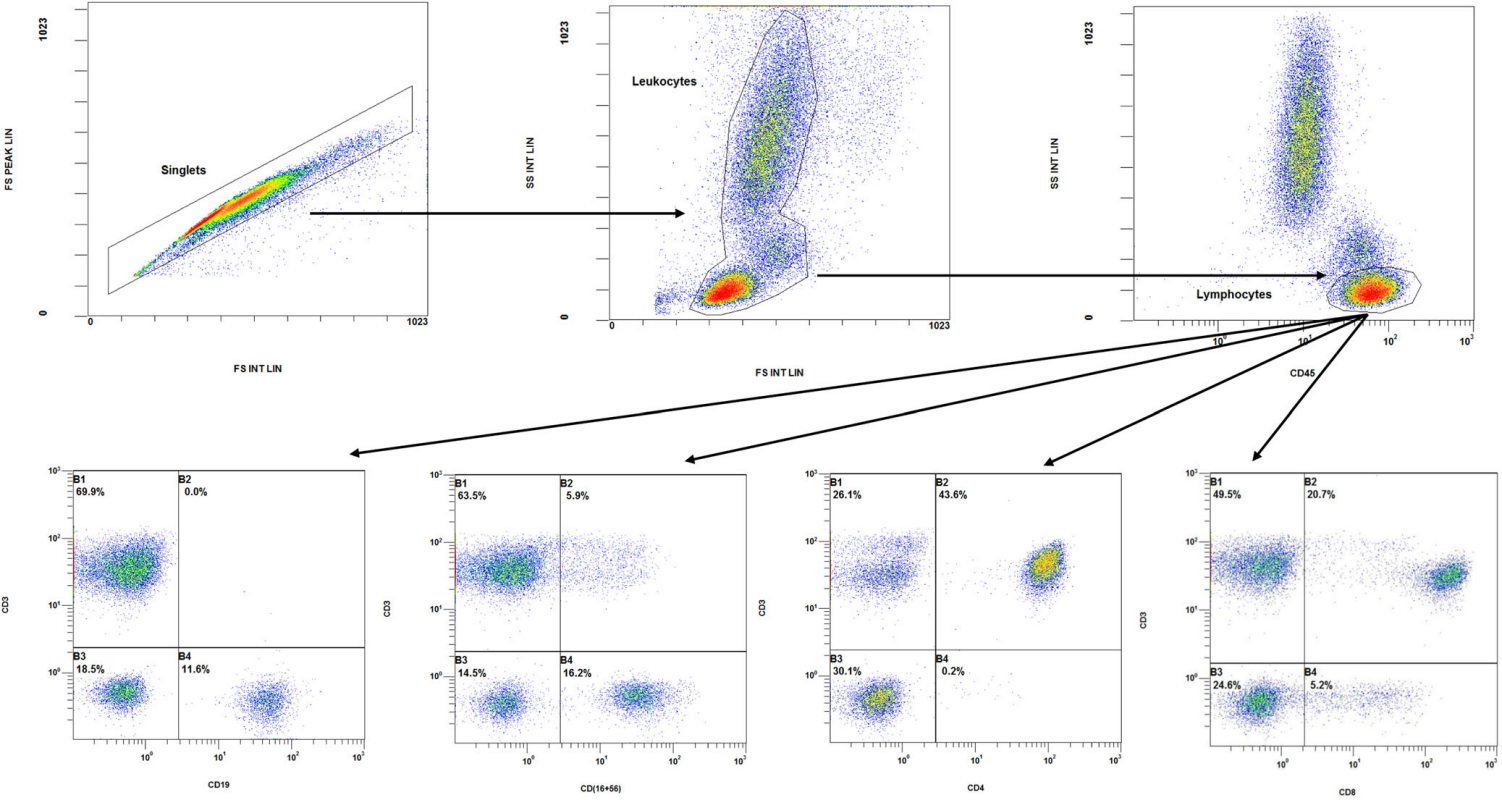
Flow cytometry analysis of immune cell population distribution. Cytometric plots with gating strategy used for immunophenotyping of CD3^−^CD19^+^, CD3^−^CD(16+56)^+^, CD3^+^CD(16+56)^+^, CD3^+^CD4^+^, CD3^+^CD8^+^ cells from peripheral blood lymphocytes.

In each case, more than 4000 events were obtained in the lymphocyte gate, characterized by high expression of CD45 and low side-scatter complexity (SSC). Fig. 4 shows the gate strategy with a dot plot, which starts by eliminating doublets (cells passing the interrogation point in groups), followed by a plot removing artifacts and detritus (forward scatter vs. side scatter) and from which the lymphocyte population was selected. Plots were generated from this lymphocyte region by combining two of the antigens expressed on the cell surface with separation by quadrants, allowing identification and determination of the percentage of subpopulations. Absolute counts of lymphocyte subpopulations were calculated by dual platform, from percentages obtained by flow cytometry and lymphocyte counts obtained by hematological analyzer using the equation: absolute count (cells/μL) = lymphocyte count (cell number/μL of the blood count)×proportion of the cell subpopulation of interest/100.

### Cytokine assays

For the determination of baseline unstimulated cytokine production, 0.25 mL of heparinized whole blood was added to 0.75 mL of RPMI 1640 medium supplemented with 10% heat-inactivated fetal bovine serum, 10 mM HEPES buffer, 2 mM l-glutamine, 50 mg of gentamicin/mL, 100 U of penicillin/mL, 100 mg of streptomycin/mL, and 0.25 mg of amphotericin B/mL and incubated at 37 °C and 5% CO_2_ for 24 h. Samples were then centrifuged at 900 × *g* for 4 min at 4 °C, following which the supernatants were collected and stored at −80 °C prior to analysis of cytokine concentrations. Plasma cytokines and cytokines secreted in cell culture media were measured using ELISA commercial kits (human TNFα, IFN-*γ*, IL-1*β*, IL-4, IL-6, and IL-10 high-sensitivity ELISA kits and human IL-18 ELISA kit, Bender MedSystems, Austria) according to the manufacturer’s instructions. All cytokine assays were calibrated against the World Health Organization international standards by the kit manufacturer. The obtained values were recalculated individually taking into account the absolute number of leukocytes. Individual leukocyte counts were measured from EDTA-treated blood using Celltac alpha MEK 6318 Hematology Analyzer (Nihon Kohden, Japan).

### Statistical proceeding of data

The foregoing indices change dynamics assessment during pre-and post-flight surveys was performed using the Wilcoxon’s test, and the results were presented as medians and the boarders of interquartile ranges.

### Reporting summary

Further information on research design is available in the Nature Research Reporting Summary linked to this article.

## Supplementary information

Reporting Summary Checklist

## Data Availability

All necessary for interpretation information is included in the manuscript. Any additional information might be provided upon request by contacting the corresponding author, D. Kuzichkin, dmitry161985@mail.ru.
