# Supplementary material for: Endothelial dysfunction markers and immune response indices in cosmonauts’ blood after long-duration space flights
Source: NPJ Microgravity. 2022 Nov 2;8:46. doi: 10.1038/s41526-022-00237-0 (PMC9630277; doi:10.1038/s41526-022-00237-0)
Supplement: Supplementary file 1 — Reporting Summary Checklist [file 41526_2022_237_MOESM1_ESM.pdf]

nature portfolio

Corresponding author(s): DBPR NPJMGRAV-00805

Last updated by author(s): Jul 19, 2022

Reporting Summary

Nature Portfolio wishes to improve the reproducibility of the work that we publish. This form provides structure for consistency and transparency in reporting. For further information on Nature Portfolio policies, see our Editorial Policies and the Editorial Policy Checklist.

Statistics

For all statistical analyses, confirm that the following items are present in the figure legend, table legend, main text, or Methods section.

n/a	Confirmed	
☐	☒	The exact sample size (n) for each experimental group/condition, given as a discrete number and unit of measurement
☐	☒	A statement on whether measurements were taken from distinct samples or whether the same sample was measured repeatedly
☐	☒	The statistical test(s) used AND whether they are one- or two-sided<br>*Only common tests should be described solely by name; describe more complex techniques in the Methods section.*
☒	☐	A description of all covariates tested
☒	☐	A description of any assumptions or corrections, such as tests of normality and adjustment for multiple comparisons
☐	☒	A full description of the statistical parameters including central tendency (e.g. means) or other basic estimates (e.g. regression coefficient) AND variation (e.g. standard deviation) or associated estimates of uncertainty (e.g. confidence intervals)
☐	☒	For null hypothesis testing, the test statistic (e.g. F, t, r) with confidence intervals, effect sizes, degrees of freedom and P value noted<br>*Give P values as exact values whenever suitable.*
☒	☐	For Bayesian analysis, information on the choice of priors and Markov chain Monte Carlo settings
☒	☐	For hierarchical and complex designs, identification of the appropriate level for tests and full reporting of outcomes
☒	☐	Estimates of effect sizes (e.g. Cohen's d, Pearson's r), indicating how they were calculated

Our web collection on statistics for biologists contains articles on many of the points above.

Software and code

Policy information about availability of computer code

Data collection	Standard commercial FACSComp software was used.
Data analysis	"Statistica 10" (StatSoft, USA) was used

For manuscripts utilizing custom algorithms or software that are central to the research but not yet described in published literature, software must be made available to editors and reviewers. We strongly encourage code deposition in a community repository (e.g. GitHub). See the Nature Portfolio guidelines for submitting code & software for further information.

Data

Policy information about availability of data

All manuscripts must include a data availability statement. This statement should provide the following information, where applicable:

- Accession codes, unique identifiers, or web links for publicly available datasets
- A description of any restrictions on data availability
- For clinical datasets or third party data, please ensure that the statement adheres to our policy

All necessary for interpretation information is included to the manuscript. Any additional information might be provided upon request.

Human research participants

Policy information about studies involving human research participants and Sex and Gender in Research.

Reporting on sex and gender	All subjects were males
Population characteristics	37-60 y.o., healthy
Recruitment	Cosmonauts who took up space missions of more than 100-days duration
Ethics oversight	Biomedicine Ethics committee of the RF SRC-Institute of Biomedical Problems, Russian Academy of Sciences/ Physiology Section of the Russian Bioethics Committee Russian Federation National Commission for UBESCO/

Note that full information on the approval of the study protocol must also be provided in the manuscript.

Field-specific reporting

Please select the one below that is the best fit for your research. If you are not sure, read the appropriate sections before making your selection.

☒ Life sciences ☐ Behavioural & social sciences ☐ Ecological, evolutionary & environmental sciences

For a reference copy of the document with all sections, see nature.com/documents/nr-reporting-summary-flat.pdf

Life sciences study design

All studies must disclose on these points even when the disclosure is negative.

Sample size	not applicable
Data exclusions	not applicable
Replication	not applicable
Randomization	not applicable
Blinding	not applicable

Reporting for specific materials, systems and methods

We require information from authors about some types of materials, experimental systems and methods used in many studies. Here, indicate whether each material, system or method listed is relevant to your study. If you are not sure if a list item applies to your research, read the appropriate section before selecting a response.

Materials & experimental systems

n/a	Involved in the study	
☐	☒	Antibodies
☒	☐	Eukaryotic cell lines
☒	☐	Palaeontology and archaeology
☒	☐	Animals and other organisms
☒	☐	Clinical data
☒	☐	Dual use research of concern

Methods

n/a	Involved in the study	
☒	☐	ChIP-seq
☐	☒	Flow cytometry
☒	☐	MRI-based neuroimaging

Antibodies

Antibodies used	For immunophenotyping commercially available fluorochrome conjugated monoclonal antibodies (eBioscience, USA) were used at the manufacturer's recommended concentrations: anti-CD45 (Clone 61D3, FITC, ref no. 11-2459-42), anti-CD19 (Clone HIB19, PE, ref no. 12-0199-80), anti-CD3 (Clone UCHT1, FITC, ref no. 11-0038-42), anti-CD4 (Clone RPA-T4, PE, ref no. 12-0049-42), anti-CD8 (Clone RPA-T8, PE, ref no. 12-0088-80), anti-CD25 (Clone BC96, PE, ref no. 12-0259-80), anti-CD11b (Clone ICRF44, FITC, ref no. 11-0118-42), anti-CD16 (Clone eBioCB16 (CB16), PE, ref no. 12-0168-42), anti-CD56 (Clone TULY56, PE, ref no. 12-0566-42), anti-HLA-DR (Clone LN3, PE, ref no. 12-9956-42), mouse IgG1 kappa isotype control (Clone P3.6.2.8.1, FITC, ref no. 11-4714-81), and mouse IgG1 kappa isotype control (Clone P3.6.2.8.1, FITC and PE, ref no. 11-4714-81 and 12-4714-42). The monoclonal antibodies used in

this study for each of the cosmonauts before and after space flight were purchased at the same time and were from the same lot number.

Validation

eBioscience antibodies are currently undergoing a rigorous two-part testing approach. To ensure that an antibody binds to the correct target, eBioscience test antibodies using one or more of the following four Thermo Fisher Scientific specificity pillars:
• Genetic modification where antibodies are tested using cell lines following knockdown or knockout technologies
• Independent antibody verification in which measurement of target expression is performed using two differentially raised antibodies recognizing the same protein target
• Functional or orthogonal testing in which functional activity or changes of target levels are measured in different cells or following cell treatments
• IP-mass spectrometry, where antibody targets are identified by mass spec following isolation by immunoprecipitation

Flow Cytometry

Plots

Confirm that:

☒ The axis labels state the marker and fluorochrome used (e.g. CD4-FITC).

☒ The axis scales are clearly visible. Include numbers along axes only for bottom left plot of group (a 'group' is an analysis of identical markers).

☒ All plots are contour plots with outliers or pseudocolor plots.

☒ A numerical value for number of cells or percentage (with statistics) is provided.

Methodology

Sample preparation

Peripheral blood was sampled by antecubital venous puncture, collected in vacutainer tubes containing ethylenediaminetetraacetic acid (EDTA) as anticoagulant (Vacuette®, Greiner bio-one, Kremsmünster, Austria), and processed by flow cytometry within two hours after sampling. For peripheral blood analysis, 100 µl of blood was added to the appropriate tubes and cells were processed according to the manufacturer's instructions. In brief, blood was incubated with the antibodies for 20 min in the dark, followed by red blood cell lysis using OptiLyse B lysing solution (Beckman Coulter, USA) for 15min in the dark. Cells were then washed twice in PBS and fixed in 200mL of IOTest3 Fixative Solution (Beckman Coulter, USA).

Instrument

BD FACSCalibur flow cytometer (BD Biosciences)

Software

CELLQuest™ software, version 5.1, for acquisition and analysis The FACStation Data Management system featuring a Macintosh® computer and CELLQuest™ software, a general purpose acquisition and analysis software program designed specifically for Becton Dickinson Immunocytometry Systems flow cytometers.

Cell population abundance

25,000 events were analyzed per tube. Isotypic controls were used for each assay to determine nonspecific staining. The fluorescence compensation was performed using CaliBRITE beads (Becton Dickinson, USA) and FACSComp software. In each case, more than 4,000 events were obtained in the lymphocyte gate, characterized by high expression of CD45 and low side-scatter complexity (SSC).

Gating strategy

In the section «Materials and methods» Figure 1 shows the gating strategy with a dot plot, which starts by eliminating doublets (cells passing the interrogation point in groups), followed by a plot removing artifacts and detritus (forward scatter vs. side scatter) and from which the lymphocyte population was selected.

☒ Tick this box to confirm that a figure exemplifying the gating strategy is provided in the Supplementary Information.

Кузичкин Д.С.
Kuzichkin D.S. [signature] 01.08.2022
